# Early identification of a COVID-19 outbreak detected by wastewater surveillance at a large homeless shelter in Toronto, Ontario

**DOI:** 10.17269/s41997-022-00696-8

**Published:** 2022-09-26

**Authors:** Samantha Akingbola, Reisha Fernandes, Susan Borden, Kimberley Gilbride, Claire Oswald, Sharon Straus, Amir Tehrani, Janis Thomas, Rebecca Stuart

**Affiliations:** 1 Toronto Public Health, Toronto, ON Canada; 2 Department of Family Medicine, McMaster University, Hamilton, ON Canada; 3 Department of Chemistry and Biology, Toronto Metropolitan University, Toronto, ON Canada; 4 Department of Geography and Environmental Studies, Toronto Metropolitan University, Toronto, ON Canada; 5 Li Ka Shing Knowledge Institute, St. Michael’s Hospital, Toronto, ON Canada; 6 Department of Medicine, University of Toronto, Toronto, ON Canada; 7 Ontario Ministry of the Environment and Parks, Toronto, ON Canada

**Keywords:** COVID-19, Wastewater, Vulnerable populations, Public health, COVID-19, eaux usées, populations vulnérables, santé publique

## Abstract

**Setting:**

Toronto (Ontario, Canada) is a large urban centre with a significant population of underhoused residents and several dozen shelters for this population with known medical and social vulnerabilities. A sizeable men’s homeless shelter piloted a facility-level SARS-CoV-2 wastewater surveillance program.

**Intervention:**

Wastewater surveillance was initiated at the shelter in January 2021. One-hour composite wastewater samples were collected twice weekly from a terminal sanitary clean-out pipe. The genetic material of the SARS-CoV-2 virus was extracted from the solid phase of each sample and analyzed using real-time qPCR to estimate the viral level. Wastewater results were reported to facility managers and Toronto Public Health within 4 days.

**Outcomes:**

There were 169 clients on-site at the time of the investigation. Wastewater surveillance alerted to the presence of COVID-19 activity at the site, prior to clinical detection. This notification acted as an early warning signal, which allowed for timely symptom screening and case finding for shelter managers and the local health unit, in preparation for the declaration of an outbreak.

**Implications:**

Wastewater surveillance acted as an advanced notification leading to the timely deployment of enhanced testing prior to clinical presentation in a population with known vulnerabilities. Wastewater surveillance at the facility level is beneficial, particularly in high-risk congregate living settings such as shelters that house transient populations where clinical testing and vaccination can be challenging. Open communication, established individual facility response plans, and a balanced threshold for action are essential to an effective wastewater surveillance program.

## Résumé

**Lieu:**

Toronto (Ontario, Canada) est un grand centre urbain qui compte une importante population de résidents mal logés et plusieurs douzaines de refuges pour cette population aux vulnérabilités médicales et sociales connues. Un assez gros refuge pour hommes sans-abri a mis à l’essai dans ses installations un programme de surveillance des eaux usées pour le SRAS-CoV-2.

**Intervention:**

La surveillance des eaux usées du refuge a commencé en janvier 2021. Des échantillons composites d’une heure ont été prélevés deux fois par semaine à partir d’un regard de nettoyage à l’extrémité du drain sanitaire. Le matériel génétique du virus du SRAS-CoV-2 a été extrait du support solide de chaque échantillon et analysé par PCR quantitative en temps réel pour estimer le niveau du virus. Les résultats des eaux usées ont été déclarés aux gestionnaires du refuge et à Santé publique Toronto dans un délai de quatre jours.

**Résultats:**

Il y avait 169 usagers sur place au moment de l’enquête. La surveillance des eaux usées a averti de la présence d’une activité de la COVID-19 sur les lieux avant sa détection clinique. Cet avertissement a servi de signal d’alerte précoce, ce qui a permis aux gestionnaires du refuge et au bureau de santé local de procéder au dépistage rapide des symptômes et à la recherche des cas en préparation pour la déclaration d’une éclosion.

**Conséquences:**

La surveillance des eaux usées a servi de notification préalable et entraîné le déploiement opportun d’un dépistage complémentaire avant la manifestation clinique dans une population qui présente des vulnérabilités connues. La surveillance des eaux usées d’une installation est avantageuse, surtout dans des milieux d’hébergement collectif à risque élevé comme les refuges qui hébergent des populations de passage, où le dépistage clinique et la vaccination peuvent être difficiles. Une communication ouverte, des plans d’intervention établis pour chaque installation et un seuil d’intervention équilibré sont essentiels à l’efficacité d’un programme de surveillance des eaux usées.

## Setting

The city of Toronto is a large urban centre with a population of 2.9 million (Government of Ontario, 2021a). The COVID-19 pandemic has had a significant impact on the overall health and well-being of Toronto residents, and this is disproportionately felt by those who are underhoused. During the first wave of the pandemic in Toronto, the estimate of COVID-19 prevalence was above 8% among the underhoused population, although the range varied across sites, reaching 70% in some facilities (Luong et al., 2022). At a baseline, persons experiencing homelessness face a multitude of social, economic, and structural barriers to equitable health care access. This diverse group of individuals experience higher levels of chronic disease, mental health concerns, and substance misuse as compared with the community at large (Guirguis-Younger et al., 2014). The COVID-19 pandemic has magnified these disparities and created further inequities for this population (Baral et al., 2021).

As of April 2021, there are over 7000 people experiencing homelessness in Toronto; 90% of these individuals live in traditional shelter settings, while the remainder are residing in encampments and other outdoor settings. Further, since 2018, there has been an estimated increase of 1100 individuals accessing the shelter system, as well as an additional 200 living outdoors (City of Toronto, 2021).

At the outset of the pandemic, a swift transformation of shelter facilities was required to accommodate an urgent need to create safer spaces for clients and staff. Most pressing was the need to reduce the number of individuals in each facility to provide safe physical distancing. As a result, 26 new temporary shelters and 24-h drop-ins were opened (City of Toronto, 2022b). This included the leasing of several hotels for improved physical distancing of 2 m in accordance with a directive from the Shelter, Support and Housing Administration (SSHA, 2020).

Vaccination is a key component in reducing the risk of severe illness due to COVID-19 infection. As those experiencing homelessness are less likely to receive regular health care, their potential for vaccination opportunities is decreased. There may be an inherent mistrust of vaccinations or of healthcare providers in general (Castillo et al., 2021). Practical challenges such as lack of health cards and mobility of clients in and out of the shelter system had to be considered when planning a mass vaccination campaign for this population. In February 2021, the City of Toronto initiated a vaccination campaign across all shelter facilities in the city. However, despite these initiatives, vaccination uptake remains lower among the underhoused population than among the general public (City of Toronto, 2022a).

Throughout the pandemic, clinical testing has been used to identify cases within the community or a facility setting. However, clinical testing is voluntary and requires cases to actively seek testing. In some instances, there may be an unwillingness to be tested and this may be particularly pronounced in shelter settings. Additionally, COVID-19 infections can be asymptomatic and there may be limited access to testing in some locations. As a result, complementary data can help to fully understand the extent of infections, and this includes testing sewage for SARS-CoV-2 signals.

Since the early days of the pandemic, the application of wastewater surveillance for tracking COVID-19 has grown (Ahmed et al., 2020; Hata et al., 2021; Hemalatha et al., 2021; Medema et al., 2020; Peccia et al., 2020; Trottier et al., 2020; Weidhaas et al., 2021). This method has previously been used to track infection rates in communities, notably for diseases such as typhoid fever (Moore, 1951) and polio (Asghar et al., 2014; Chen, 2020; Trask & Paul, 1942). Wastewater surveillance has been shown to be an effective tool for tracking COVID-19 by providing an early warning of new or emerging cases that complements clinical testing efforts (Zhu et al., 2021), as well as monitoring current trends. Wastewater surveillance for COVID-19 involves testing raw wastewater to isolate and detect SARS-CoV-2 viral RNA shed from both symptomatic and asymptomatic infected people. Wastewater can be sampled at a wastewater treatment plant to achieve broad population coverage (Fitzgerald et al., 2021), upstream in the sewershed to target specific communities to assess relative risk (Yeager et al., 2021), and at individual facilities (e.g., schools, hospitals, long-term care homes) to provide more detailed and targeted information for health authorities (Wong et al., 2021). At facilities, this information can then be used in combination with clinical data to identify outbreaks, enhance testing on-site, and implement vaccination clinics.

Several facilities in downtown Toronto were included in a wastewater surveillance program to monitor and detect SARS-CoV-2. The objective of this program was to determine if wastewater could be used as an early warning signal to identify potential outbreaks in the absence of mass clinical testing of all individuals regardless of symptom status. One of those facilities, a large homeless shelter, was found to have positive wastewater signals in late August 2021, despite having no known clinical cases. This paper describes how the samples were obtained, analyzed, reported, and responded to by Toronto Public Health (TPH) and the shelter. 

## Intervention

Toronto Metropolitan University (TMU, formerly known as Ryerson) began a COVID-19 wastewater surveillance program in the fall of 2020. Sampling sites were established at a City of Toronto wastewater treatment plant and in six communities in the west end of the city. In January 2021, wastewater sampling was expanded to multiple facility sites, including several hospitals, long-term care homes, and shelters.

At this shelter site, in collaboration with the facilities management team, TMU identified the terminal sanitary sewer clean-out for the entire shelter. The sewer clean-out for the shelter connects to the lateral sewer line and subsequently to the municipal combined sewer. To collect wastewater, a sample intake line was lowered into the clean-out pipe until the end was sitting in flowing sewage in the lateral pipe. An Avalanche® refrigerated autosampler (Teledyne ISCO, NE, USA) was programmed to pump out sewage once every 10 min for 1 h. Collection of the sample was repeated twice per week (Tuesday and Thursday) between 8:30 a.m. and 9:30 a.m. when sewage flows were relatively high.

Wastewater samples were transported to the lab within 1 h of collection and stored at 4°C for a maximum of 24 h prior to processing. Processing involved centrifugation of the samples and extraction of RNA from approximately 100–150 mg using the RNeasy PowerMicrobiome extraction kit (Qiagen, MD, USA). The RNA was used for RT-qPCRs targeting the N1 and N2 regions of the SARS-CoV-2 genome using the Reliance one-step qPCR kit (Bio-Rad, CA, USA) and protocol. Pepper Mild Mottle Virus (PMMoV) was also targeted as a fecal biomarker and used to normalize the average N1/N2 viral concentrations in each sample. Further details are provided in the Appendix.

**Fig. 1:**
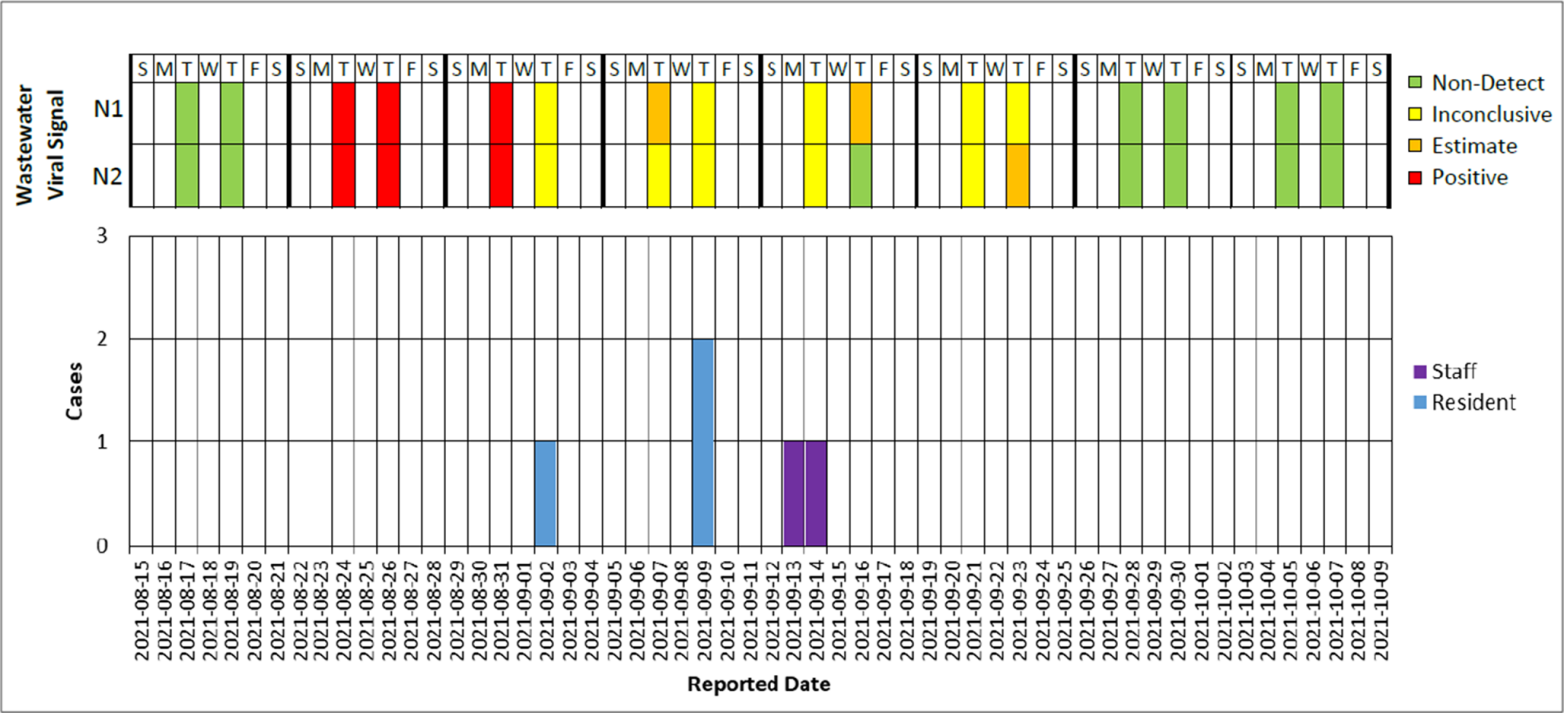
Wastewater surveillance signal (top panel) and epidemic curve of confirmed cases by role (bottom panel) at a shelter outbreak, Toronto, August 24–October 7, 2021 (*n* = 5). Non-detect: The qPCR did not produce any value. Inconclusive: The raw Ct value fell outside the linear range of the standard curve and was above the *y*-intercept of the standard curve. Estimate: The raw Ct value fell outside the linear range of the standard curve, and the value was between the *y*-intercept of the standard curve and the limit of detection (LOD). Positive: The raw Ct value was within the linear range of the standard curve. Reported date: The date the case was reported to TPH

Average concentrations of PMMoV and SARS-CoV-2 RNA for the N1 and N2 targets were obtained from the three technical replicates. The average N1 and N2 viral concentrations were averaged and normalized to PMMoV to account for variability in the fecal content of each sample. A sample was considered positive when both N1 and N2 PCR targets were greater than the LOD. The footnotes under Fig. 1 provide further definitions of wastewater viral signal classification.

## Outcomes

In January 2021, wastewater sampling at the shelter commenced, with results reported within 4 days of sample collection, twice per week, to TPH and shelter managers. Reported results included a flag to indicate if the final normalized SARS-CoV-2 copy numbers were above or below the LOD of the analysis.

On August 27, 2021, the facility and TPH were notified by TMU of the first positive wastewater result from a sample collected on August 24 (Fig. 1). A plan was then implemented for the shelter to confirm that no one on-site was symptomatic, while awaiting confirmation of the second wastewater sample. Confirmation of no symptomatic staff or clients was given to TPH on August 30. Due to the absence of symptomatic individuals at the time, shelter management was not aware of any COVID-19 activity on the site prior to these wastewater notifications. On August 30, TPH was notified that the second wastewater sample from August 26 was positive. This second positive result led to the development of an enhanced testing plan on August 30 to test all unvaccinated clients and staff, as well as all new admissions within the past 2 weeks who had not tested positive within the previous 90 days. Last, as part of the routine practice throughout the pandemic, all symptomatic individuals in shelters continued to be required to undergo testing regardless of their vaccination status. An investigation was initiated by TPH on August 31 in response to the positive wastewater signals. The first clinical case was reported to TPH on September 2, 2021, and an outbreak was declared on that same day. Additional clinical cases were reported on September 9, 13, and 14.

There were several baseline measures in place at the shelter, including symptomatic testing, regular cleaning, physical distancing, and contact tracing. The notification of two consecutive positive wastewater signals led to the escalation of enhanced testing to identify the individual active cases. Further public health measures following the identification of cases and the declaration of an outbreak included cohorting, separate isolation units for close contacts and confirmed cases, and risk stratification (identifying high-risk clients and providing them with the necessary resources and support). Additional measures included further enhanced sanitization and environmental cleaning.

While the facility has an overall capacity of 500 beds and 300 staff, there were only 169 shelter residents and 105 staff on-site at the start of the investigation. There were a total of five confirmed cases and 11 close contacts identified as part of this outbreak. Two of the confirmed cases were staff members, and the remaining three were shelter residents (Table 1). Four of the five cases were symptomatic, with onset dates ranging from September 1 to September 11, 2021. The cases ranged in age from 34 to 76 years, with a median of 60 years. Of the five cases, two were fully vaccinated (two doses) breakthrough cases. The reported dates of the five cases ranged across 12 days, and after approximately 2 weeks with no further cases identified, the outbreak was declared over on September 26 (Fig. 1). The wastewater surveillance results returned to less than LOD (non-actionable) levels by early September, and were fully non-detectable (i.e., qPCR did not produce any value) after September 28, 2021 (Fig. 1). The presence of inconclusive results until the 23^rd^ of September is possibly due to viral shedding from cases or staff who returned to the site after their isolation, as viral shedding is known to last up to 3 weeks (Zhang et al., 2021).

**Table 1:** Demographic and clinical details of confirmed cases

Summary	Cases
Case count and demographics	*N* (%)
Number of cases	5
Male	5 (100%)
Female	0 (0%)
Age at onset	
Mean	54
Median	60
Range	34–76
Vaccination status	*N* (%)
Fully vaccinated^†^	2 (40%)
Partially vaccinated^‡^	0 (0%)
Unvaccinated^§^	3 (60%)
Unknown^††^	0 (0%)
Facility role	*N* (%)
Resident	3 (60%)
Staff	2 (40%)
Outcome	*N* (%)
Recovered	5 (100%)

^†^Fully vaccinated: individuals who, at the time of their COVID-19 diagnosis, have (1) received both doses of a two-dose Health Canada–approved COVID-19 vaccine series (i.e., dose two of two) and 14 or more days have elapsed following dose 2 administration; *or* (2) received one dose of a one-dose Health Canada–approved COVID-19 vaccine product (i.e., dose one of one) and more than 14 days have elapsed following dose 1 administration

^‡^Partially vaccinated: individuals who, at the time of their COVID-19 diagnosis, have (1) received only the first dose of a two-dose Health Canada–approved COVID-19 vaccine series and 14 or more days have elapsed following dose 1 administration *or* (2) received two doses of Health Canada–approved COVID-19 vaccine but are not yet considered fully vaccinated (i.e., less than 14 days following dose 2 administration)

^§^Unvaccinated: individuals who, at the time of their COVID-19 diagnosis, have (1) not received a dose of a Health Canada–approved COVID-19 vaccine (this includes individuals who at the time of illness were ineligible to get a COVID-19 vaccine) *or* (2) been vaccinated for COVID-19 with a Health Canada–approved vaccine, but are not yet protected from vaccination (i.e., less than 14 days following dose 1 administration)

^††^Unknown: individuals who do not have a record in Ontario’s vaccine administration system and (1) follow-up has yet to occur to request vaccination status information, or (2) case chose not to disclose their vaccination status, or (3) case is untraceable or lost to follow-up

## Implications

Wastewater surveillance proved to be a beneficial tool to complement clinical testing and surveillance in a shelter which houses transient populations, where testing can be challenging.

This study documents the first time that an outbreak investigation was initiated in a facility in Toronto using wastewater surveillance. Prior to the first positive signal, facility managers were not aware of COVID-19 activity on-site. The first positive wastewater sample triggered an assessment to determine if anyone on-site was symptomatic; the signal also allowed for facility managers and TPH to be on high alert as they began to prepare for further investigation and control measures. One positive signal indicates a fecal sample with viral RNA, which could be due to a resident or staff frequenting the facility, but could also be a transient visitor to the shelter. The second positive wastewater signal resulted in an escalation of public health measures, namely enhanced targeted testing, as two consecutive positive signals indicated continued presence of the virus and by that time a higher likelihood of identifying the individual cases with the enhanced testing protocol.

Our findings of wastewater surveillance as an early warning tool are similar to what was found in a study in Spain (Randazzo et al., 2020), where viral RNA was found in wastewater samples in different regions prior to clinical detection and reporting by health authorities.

The success of using wastewater as an innovative tool was due in part to a pre-established facility response plan in which all partners had committed to mobilize and plan for public health interventions upon receipt of positive wastewater signals. Having this pre-defined response plan for positive wastewater signals at the facility level allowed for a timely response to alerts. Groups that wish to adopt wastewater surveillance as a novel tool would benefit from having open communication between all partners, establishing individual facility response plans, and determining a balanced threshold for action. It is important to note that different types of facilities may require other plans. Depending on the setting and population, it may be preferable to enact certain protocols at the outset of the first positive signal; in other settings, such as those frequented by members of the community, facility managers may opt to wait for more than two signals before enacting protocols such as mass testing.

It is important to consider local epidemiology in the context of wastewater surveillance. This outbreak was identified as a Delta variant outbreak based on the genetic profiles of the cases’ clinical specimens, consistent with the near universal dominance of the highly transmissible Delta variant in Toronto at that time. The detection of positive wastewater results, and the subsequent declaration of an outbreak, took place during the fourth wave of the pandemic, when case counts had risen following the summer re-opening. By the time the outbreak was declared over at the end of September 2021, the weekly incidence rate of new cases in Toronto had fallen to approximately 25 per 100,000 (down from 35 per 100,000 at the end of August, when the signal was first detected in the wastewater). Detection of wastewater signals may be impacted by local epidemiology at the time of sampling, and it is possible that the elevated level of transmission at the end of August may have aided detection, despite the transient nature of the shelter population.

While wastewater surveillance was successful at the facility level in this case, there are limitations to using this tool. Environmental and weather-related factors can lead to variation in the SARS-CoV-2 viral wastewater signals (Randazzo et al., 2020). Further, positive signals may not always lead to the detection of clinically confirmed cases due to the transient nature of the population utilizing facilities, such as in shelters. Therefore, positive wastewater signals in such sites are best accompanied by expanded clinical testing in order to identify individuals and respond accordingly. An additional limitation is that a wastewater signal could be missed due to infrequent or inadequate sample collection times. In a population with high turnover, this may reduce the ability to detect whether an infected person or persons were at the facility. Ideally, a 24-h composite sample, which takes a small sample of wastewater over an entire day, would be more likely to pick up positive signals regardless of transience. However, at this site, it was not possible due to limitations in the sampling access point. To compensate for this, composite samples were collected over a 1-h period twice per week at the same time in the morning when higher flows were detectable.

Fecal shedding in wastewater can be observed prior to symptom onset, and can also continue post-recovery for several weeks. However, shedding patterns are dependent on clinical and demographic characteristics such as immune status and age (Bertels et al., 2022). Shedding can last for up to 3 weeks, and viral RNA is found in feces even after it is no longer detectable by respiratory sampling (Zhang et al., 2021). Notably, not all people infected with SARS-CoV-2 will shed in their feces; a meta-analysis estimated that approximately half of COVID-19 cases have fecal shedding (Van Doorn et al., 2020).

In our case study, the presence of inconclusive results until September 23^rd^ (Fig. 1), 3 weeks after the first symptom onset date, suggests that the staff/residents may have continued to shed for several weeks after they were diagnosed. Further work is required to understand the impact of shedding variation on wastewater surveillance. Additional initiatives to validate wastewater as a surveillance tool in outbreak detection and response are essential.

Wastewater surveillance at smaller facilities requires careful consideration regarding privacy and ethics. For others considering surveillance at facility levels, early conversations regarding data reporting should be discussed. As noted by Scassa et al. (2022), the increase in wastewater surveillance requires thorough consideration into how results are communicated due to privacy concerns and potentially negative ramifications.

At the end of 2021, the province of Ontario amended its criteria and access to clinical PCR testing (Government of Ontario, 2021a, 2021b). Given these changes, the generalizability of reported clinical data for COVID-19 surveillance is limited. As the pandemic proceeds, wastewater surveillance will be of increasing importance to monitor trends of COVID-19 activity in the community, as done by the Ontario COVID-19 Science Advisory Table (2022). On an outbreak detection level, wastewater surveillance will continue to be a helpful ancillary and complementary tool for high-risk settings such as shelters.

## Conclusion

This study demonstrates the potential usefulness of wastewater surveillance as an early warning signal prior to clinical detection of cases at a shelter. In this instance, it was a useful tool to provide additional situational awareness of COVID-19 activity in combination with traditional surveillance.

## Implications for policy and practice

What are the innovations in this policy or program?

- Application of a relatively new surveillance methodology to a rapidly changing pandemic, enabling the early detection of infectious disease activity prior to clinical presentation in a vulnerable population.
- Enabling enhanced testing to identify cases, and advanced planning for future outbreak control in an effort to prevent further spread.

What are the burning research questions for this innovation?

- Greater frequency in wastewater sampling (either temporally or spatially) to increase the likelihood of detecting signals, particularly in transient facilities such as shelters.
- A better understanding of the shedding frequency and timing of SARS-CoV-2 in the feces of infected individuals would be helpful to understanding the relationship between clinical infections and wastewater signals.
- Wastewater surveillance can be expanded to include the detection of any fecal-shed pathogen to monitor infection prevalence in a given population.

## Data Availability

Available on request

## Appendix

**Details of wastewater sampling methodology**

Processing involved the centrifugation of 200 mL of the sample for 50 min at 12,000 × g without brake. The supernatant was decanted without disturbing the solid pellet, and its wet weight was recorded. Approximately 100–150 mg of the wet pellet was used for RNA extraction using the RNeasy PowerMicrobiome extraction kit, and the process was automated with a QIAGEN QIAcube Connect instrument (Qiagen, MD, USA) to automate the RNA extraction procedure. In the final step of the RNA extraction, 100 μL of RNA-free water was used to elute the RNA. The RNA purity and concentration were measured using a NanoPhotometer™ Pearl (Implen, München, Germany). The A260/280 and A260/230 ratios for all analyzed RNA samples were between 1.9 and 2.0. The RNA was then stored at −20°C until analysis.

Primers/probe sets for SARS-CoV-2 published by the United States Centers for Disease Control and Prevention (CDC, 2021) were used in this study and were purchased as premixed kits (IDT, Coralville, IA, USA). Detection of the two targets in the nucleocapsid gene (N1 and N2) was performed by RT-qPCR in three technical replicates. Pepper Mild Mottle Virus (PMMoV) was used as a fecal biomarker and an internal amplification control. The PMMoV primers and FAM labeled probe (D’Aoust et al., 2021) were custom ordered from IDT, Coralville, IA, USA.

The qPCRs were set up following Bio-Rad’s Reliance one-step qPCR supermix (Bio-Rad, CA, USA) protocol. Briefly, 5 μL of template was used for every reaction for a total reaction volume of 10 μL. The master mix for each target was manually assembled, but the dilution series and the loading of the 384-well plate was automated by Qiagility instrument (Qiagen, MD, USA). The qPCRs were performed on Bio-Rad’s CFX384 OPUS real-time system (Bio-Rad, CA, USA) with the following thermocycler conditions: 50°C incubation for 10 min, initial denaturation at 95°C for 10 min, 45 cycles of 95°C denaturation for 30 s, and 60°C annealing/elongation for 30 s, and signals were recorded at the end of each cycle. The data were analyzed using Bio-Rad CFX Maestro 2.2 software (Bio-Rad, CA, USA), and the cycle threshold (Ct) values were automatically generated for each primer set by using the default settings set by the program.

Quality control included validation of the RT-qPCR protocol with no-template controls (NTCs) and generation of five-point standard curves for each target in triplicates. The primer efficiencies for each target ranged from 95% to 105%; the *R*^2^ value was > 0.98. The limit of detection (LOD) was calculated to be ~ 2 copies/mL with a 95% coefficient of variation.
